# Supplementary material for: Signal-processing and adaptive prototissue formation in metabolic DNA protocells
Source: Nat Commun. 2022 Jul 8;13:3968. doi: 10.1038/s41467-022-31632-6 (PMC9270428; doi:10.1038/s41467-022-31632-6)
Supplement: Supplementary file 1 — Supplementary Information [file 41467_2022_31632_MOESM1_ESM.pdf]

Supplementary information for

Signal-Processing and Adaptive Prototissue Formation in Metabolic DNA Protocells

Avik Samanta,[1*] Maximilian Hörner,[2] Wei Liu[1], Wilfried Weber,[2] Andreas Walther[1,3*]

[1]A3BMS Lab, University of Mainz, Department of Chemistry, Duesbergweg 10-14, 55128 Mainz, Germany.

[2]Faculty of Biology, Cluster of Excellence CIBSS - Centre for Integrative Biological Signalling Studies, University of Freiburg, 79104 Freiburg, Germany.

[3]Cluster of Excellence livMatS @ FIT – Freiburg Center for Interactive Materials and Bioinspired Technologies, University of Freiburg, Freiburg, Germany

*Corresponding authors.

Email: avik.samanta@uni-mainz.de, andreas.walther@uni-mainz.de

Supplementary Methods

Materials and Instrument:

ssDNA oligomers (as listed below in Table S1) were purchased from Integrated DNA Technologies (IDT) and Biomers GmbH. The enzymes, such as T_4 ligase (low concentration 4 U μL^{-1}), Exonuclease I (40 U/µL), Exonuclease III (200 U μL^{-1}), Inorganic pyrophosphatase (2 U μL^{-1}) and Φ_{29} polymerase (10 U μL^{-1}) were purchased from Lucigen (Biozyme), New England Biolabs and Promega. The deoxynucleotide triphosphates (dATP, dTTP, dGTP and dCTP) (100-110 mM 1 mL buffered solution) were purchased from Jena Bioscience. Sodium chloride (NaCl), Magnesium acetate tetrahydrate ($MgAc_2$), Citric acid, disodium ethylenediaminetetraacetate dehydrate (EDTA), Tris(2-carboxyethyl)phosphine hydrochloride, tris(hydroxymethyl)aminomethane hydrochloride (TRIS-HCl and Trizma buffer substance pH=8), bovine serum albumin (BSA) and acetic acid were purchased (as bioreagent grade if available) from Sigma-Aldrich. Ultrapure Agarose Low EEO was purchased from AppliChem. SYBR gold, loading dye, 50 bp, and 1 kb ladder were purchased from ThermoFisher Scientific. The basic solution for protocell preparation is a TE buffer that consists of 10 mM of Tris(hydroxymethyl)aminomethane (pH=8.0), 1 mM of EDTA, and 50 mM $MgCl_2$. The buffer exchange was performed according to the specific experiment. DNA sequences (in TE buffer) and the glycol-dispersed enzymes were always stored frozen at –25 °C. Gel electrophoresis was run using 1-2 wt% Agarose gel in TAE buffer containing 40 mM of TRIS-HCl, 20 mM of acetic acid, and 1 mM of EDTA. The Gamry potentiostats were used to apply and control the voltage in the gel electrophoresis experiments.

Thermal annealing and heating ramps were programmed on a Personal Thermocycler (Jena Analytics), DNA concentrations were determined using a ScanDrop (Jena Analytic, FlashSoftPRO v1.2) spectrophotometer with a standard value of 33 $\mu g/OD_{260}$. Confocal laser scanning microscopy (CLSM) was performed on Leica Stellaris 5 microscope (LasX v4.3.0.24308) with four Laser lines and three HyD detectors. The temperature of the sample during the microscopic experiments was maintained with a TOKAI HIT temperature-controlled device. The time-dependent, temperature-controlled spectrofluorometric measurements were performed using a TECAN (SPARK control v3.1) microplate plate reader. Flow cytometry experiments were performed on a Gallios flow cytometer (Beckman Coulter, Brea, CA).

Supplementary Note

Abbreviations

PC/PCs	Protocell/protocells
Dz	DNAzyme
ssDNA	Single-stranded DNA
dsDNA	Double-stranded DNA
[r.u.]	Concentration of the repeating unit of the ssDNA multiblock polymer
pA	polyadenine
pT	Polythymine
Dz⊂PCs	DNAzyme-encapsulated protocells
FRET	Fluorescence (or Förster) resonance energy transfer
DSD	Dynamic DNA strand displacement
LLPS	Liquid-liquid phase separation
BHQ	Black-hole quencher
DCBC	DNAzyme-catalyzed bond cleavage
BCDM	Bond-cleavage driven duplex melting
CLSM	Confocal laser scanning microscopy

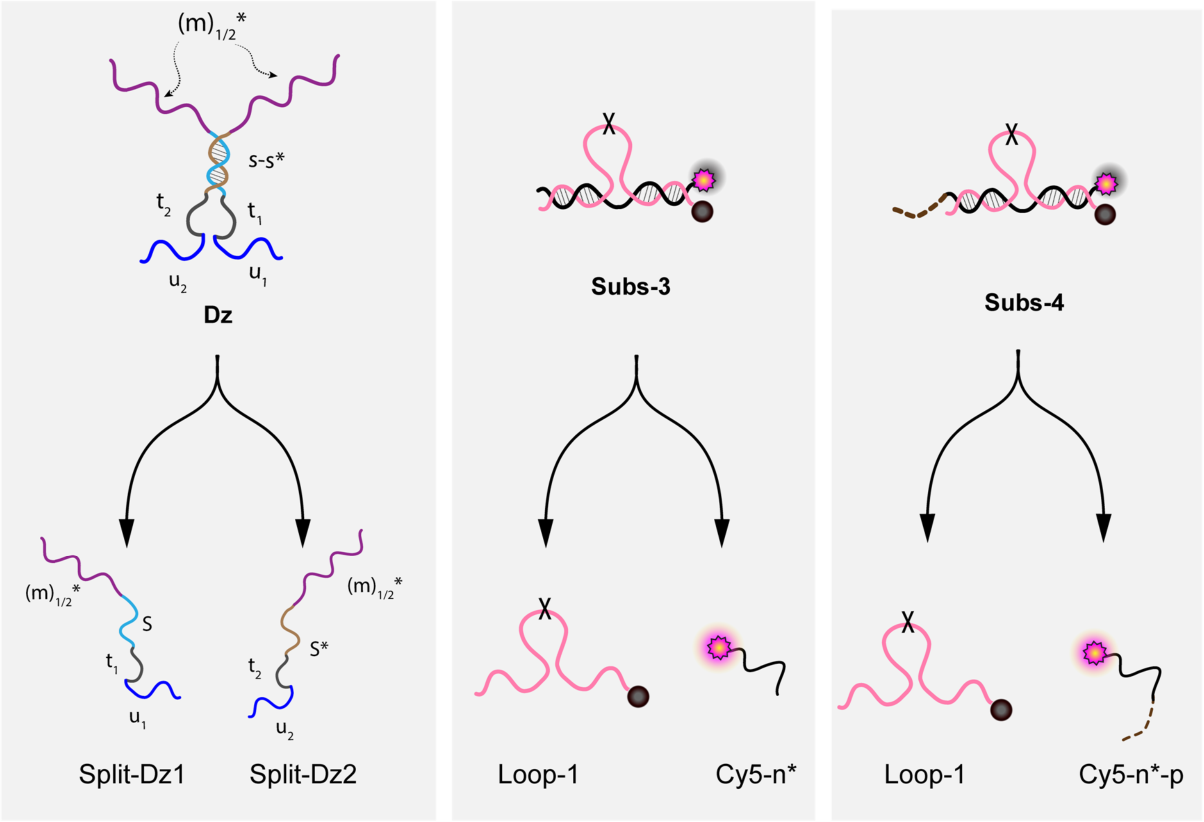

Supplementary Figure 1: Schematic representation of the DNAzyme (Dz) and two loop substrates with constitutional ssDNA oligomers. The detailed sequences are listed in Supplementary Table S2.

Supplementary Table 1. The repeating unit sequences of multiblock polymers, p(A_{20}-m) and p(T_{20}-n).

p(A_{20}-m)	(A_{20}-TTAGGATAGATATACGGGTTC)$_{25\text{-}47}$
p(T_{20}-n).	(T_{20}-GATTTTAGAGGATCGTGTGGTTTTAC)$_{20\text{-}40}$

Supplementary Table 2. Sequences used, with their abbreviations, the sequence codes used for ordering at IDT, the purification grade and modifications.

	Name	Sequence 5'→3'	Purification	Modification
**Templates**	Temp(A_{20}-m)	/5Phos/ATC TAT CCT AAT TTT TTT TTT TTT TTT TTT TGA ACC CGT AT	HPLC	5'-Phosphorylation
	Temp(T_{20}-n)	/5Phos/ ATC CTC TAA AAT CAA AAA AAA AAA AAA AAA AAG TAA AAC CAC ACG	HPLC	5'- Phosphorylation
**Ligation**	m	TTA GGA TAG ATA TAC GGG TTC	HPLC	None
	n	TTT TAG AGG ATC GTG TGG TTT T	HPLC	None
**Primers**	m-exo	TTA GGA TAG ATA TAC GGG T*T*C	Desalting	Phosphorothioated Twice
	n-exo	TTT TAG AGG ATC GTG TGG TT*T* T	Desalting	Phosphorothioated Twice
**Barcode* strands**	$Atto_{488}$-m*	/5ATTO488N/ TGA ACC CGT ATA TCT ATC CTA A	HPLC	5' Atto 488 (NHS ester)
	Cy5-n*	/5Cy5/ AAA ACC ACA CGA TCC TCT A	HPLC	5' Atto 565 (NHS ester)
	Cy5-n*-p	/5Cy5/ AAA ACC ACA CGA TCC TCT ACT CGA G	HPLC	5' Atto 565 (NHS ester)
	$Atto_{488}$-n_{short}*	/5ATTO488/ ACC ACA CGA TC	HPLC	5' Atto 647N (NHS ester)
	$Atto_{565}$-n_{short}*	/5ATTO565/ ACC ACA CGA TC	HPLC	5' Atto 647N (NHS ester)
	Dz-base (m)	AAA ATTA GGA TAG ATA TAC GGG TTC AAA	Desalting	None
	n*	AAA ACC ACA CGA TCC TCT AAA A	Desalting	None
**DNAzyme strands**	Split-Dz1	TTG AAC CCG TAT AGTA CTA TGC ACA CCA TGT TGA AGA	HPLC	None
	Split-Dz2	TAG CTG AGC GAT TGC ATA GTA CTCT ATC CTA ATT T	HPLC	None
**Substrate strands**	Subs-1	/5Cy5/TCT TCA TrAG CAG CTA/3IAbRQSp/	HPLC	5' Cy5 and 3' Iowa Black quencher (with phosphodiester bonds)
	Subs-2	/5Cy3/TCT TCA TrAG CAG CTA/3IABkFQ/	HPLC	5' Cy3 and 3' Iowa Black quencher (with phosphodiester bonds)
	Loop-1	AGT TAT AGG ATA TCA TrAG CAG TTC GTG TAG TT/3IAbRQSp/	HPLC	3' Iowa Black quencher (with phosphodiester bonds)

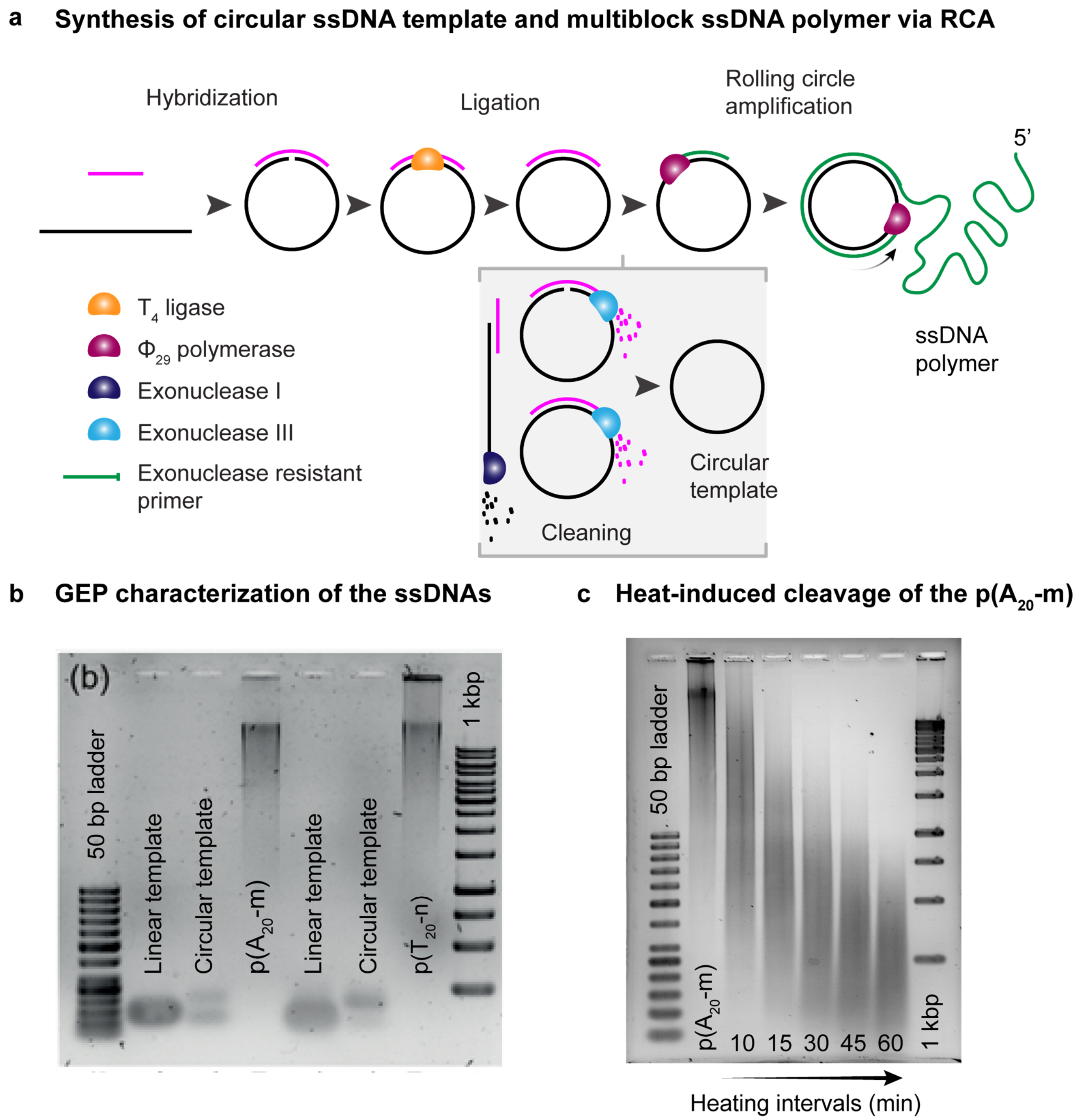

Supplementary Figure 2: ssDNA synthesis via rolling circle amplification (RCA). (a) Schematic representation of the complete process and (b) corresponding gel electrophoresis. A commercial ssDNA (black) is circularized using a complementary ligation strand (magenta) and ligated using T_4 ligase. The non-ligated products and ligation strands are then digested by Exonucleases I and III, and the purified templates are amplified using a primer Φ_{29} polymerase (maroon). (b) Gel electrophoresis characterization of the linear ssDNA templates, circular templates, and the RCA products, namely p(A_{20}-m) and p(T_{20}-n). (c) Gel electrophoresis of the thermal cleavage of p(A_{20}-m) at 95°C in TE buffer together with the linear template p(A_{20}-m). Gel electrophoresis is run in TAE buffer using 1 % agarose gel and 6 V/cm for 90 min with SYBR gold staining. 50 bp Ladder bands correspond to 50, 100, 150, 200, **250**, 300, 400, **500**, 600, 700, 800, 900 and 1000 base pairs, and the 1kbp ladder bands correspond to 250, 500, 750, **1000**, 1500, 2000, 2500, **3000**, 3500, 4000, 5000, **6000**, 8000 and 10000 base pairs from top to bottom.

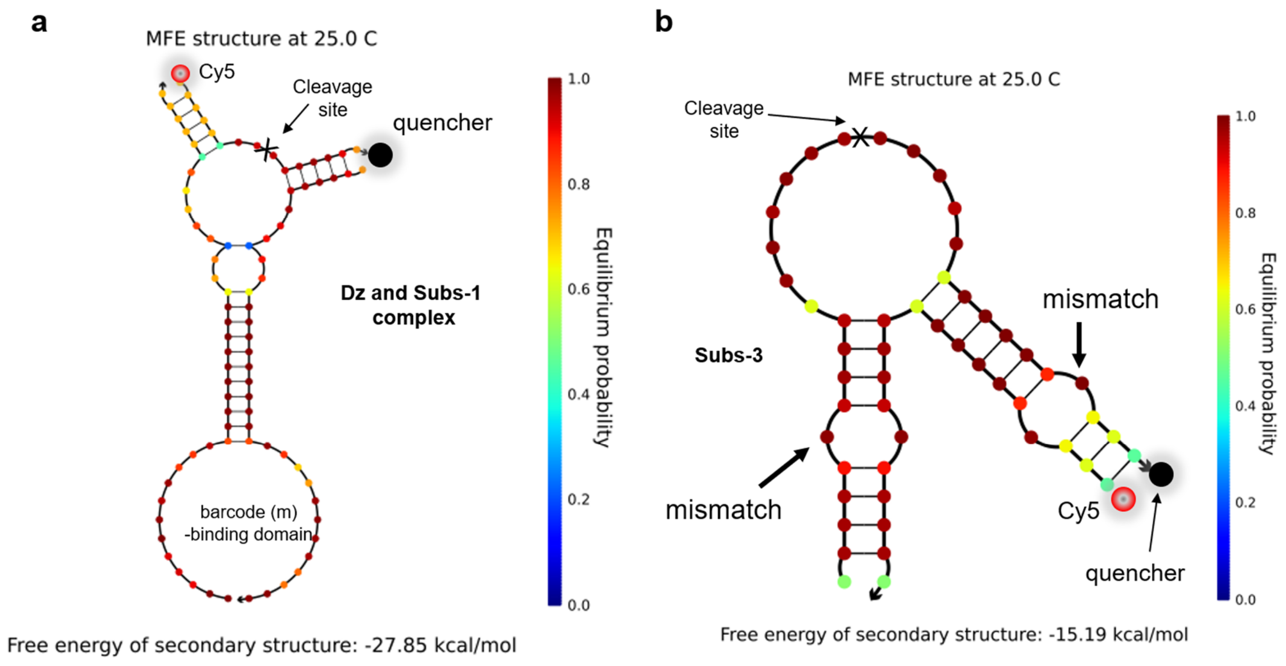

Supplementary Figure 3: NUPACK[1] simulated structures of Dz/Subs-1 complex and Subs-3 at 25 °C. The sequences were optimized using melting temperatures of different domains, and mismatches are included to facilitate release upon catalytic bond cleavage.

DNAzyme catalysis and stoichiometry in PC

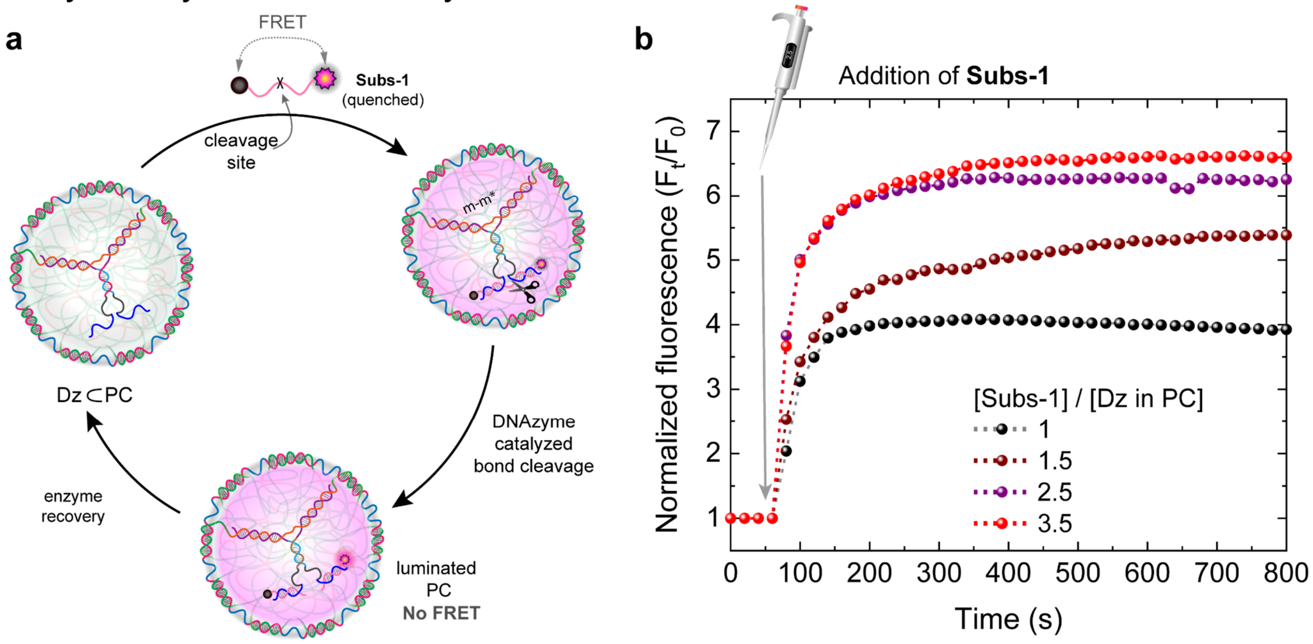

Supplementary Figure 4: Intra-protocellular catalytic cleavage of self-reporting substrate and the stoichiometric ratio. a) Schematic representation of a catalytic cycle for RNA-linkage cleavage in Subs-1 in PC interior and fluorescence enhancement (magenta) upon uncaging the Cy5-appended product. b) Time-dependent spectrofluorimetric investigation of DCBC in PC at various [Subs-1] to [Dz in PC] stoichiometry. It is observed that above 2.5 equivalent of the substrate, the catalysis is poisoned by the product's stickiness at the active catalytic site at 25°C. [Dz⊂PCs] = 2 μM in TE buffer at pH 8.

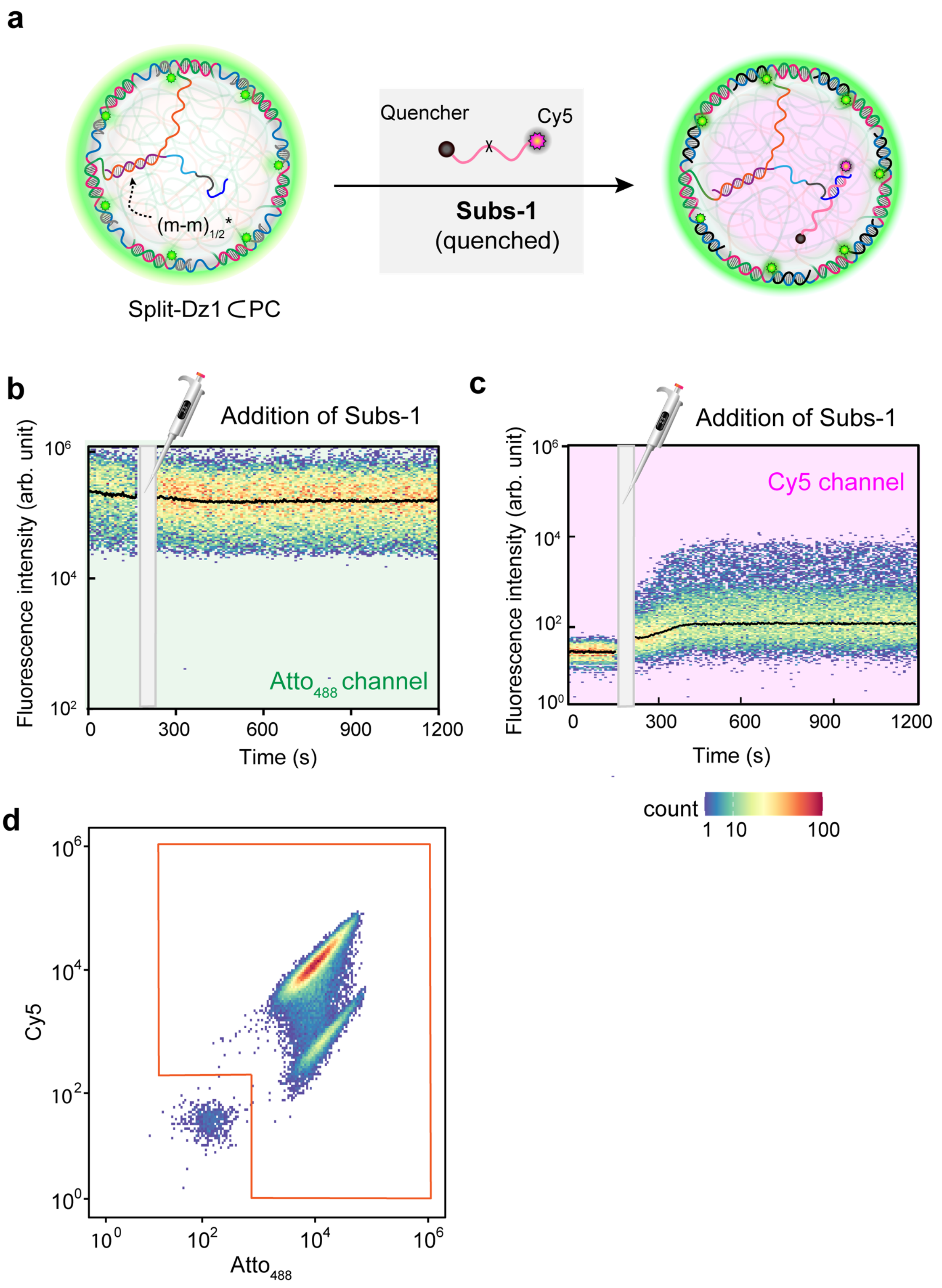

Supplementary Figure 5: Protocellular (control experiment) Atto488 and Cy5 fluorescence were monitored over time by flow cytometry. a) Schematic representation of dormant PC (shell labeled with Atto488-n*, green) and Subs-1 binding. b) and c) The time-dependent fluorescence intensity of Atto488 and Cy5 of each PC is measured using flow cytometry. The slight increase at the Cy5 channel (magenta) represents the residual fluorescence of the quenched Subs-1. d) The gating of the PC population concerning two fluorescence channels for the kinetics, presented in Figures 2f and g. The line shows the moving median. The green, and magenta channels correspond to Atto488 and Cy5, respectively. Condition: [Split-Dz1⊂PC] = 0.5 µM and [Subs-1] = 1.3 µM in TE buffer at pH 8.

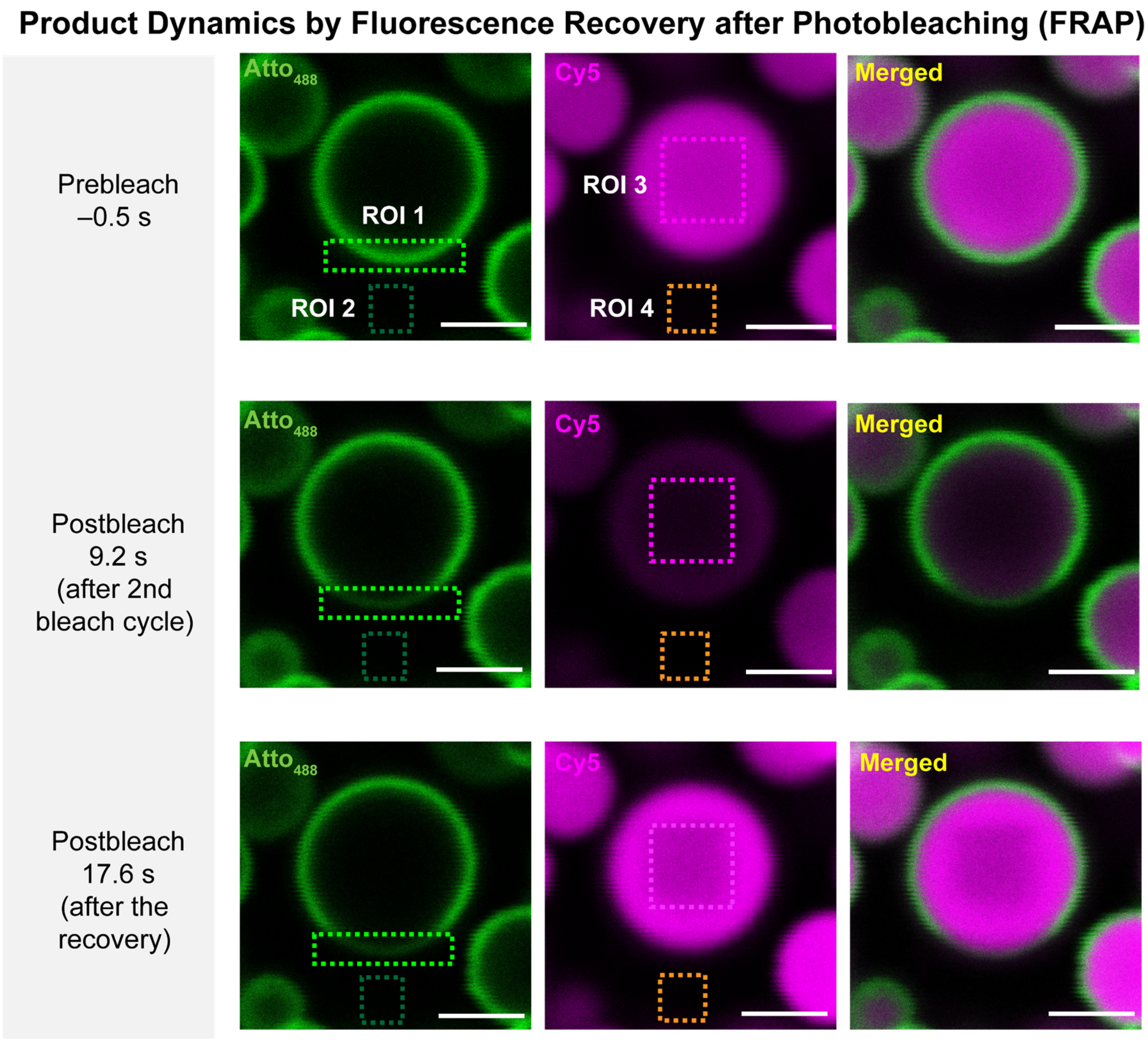

Supplementary Figure 6: The Dz-catalyzed product dynamics in the core of PC using FRAP. CLSM image of a Dz⊂PC after the DCBC of Subs-1 and the photo-bleaching is performed within the dotted rectangles (respective ROIs). Three rows of CLSM images depict the pre-bleach state, the post-bleach state immediately after the second bleach cycle, and another post-bleach state after the recovery, hence just before the third bleaching cycle. The entire bleach and post-bleach cycles are represented in Supplementary Video S1. The corresponding fluorescence recovery plots from ROI-1 to 4 are shown in Figure 3b and c. The green, and magenta channels correspond to $Atto_{488}$ and Cy5, respectively. Scale bars: 5 µm.

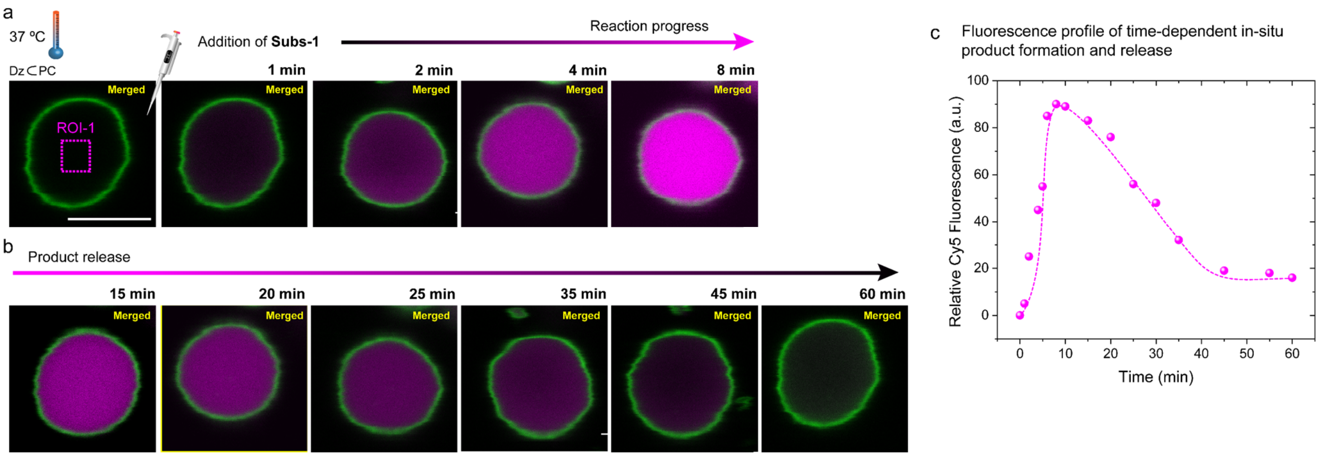

Supplementary Figure 7: Dz-catalyzed intraprotocellular product formation and the product release. a) Time-dependent CLSM images representing the bond cleavage of Subs-1 by the encapsulated Dz in the PC core. The shell of Dz⊂PC is labeled with $Atto_{488}$-n* (green), and the evolving magenta fluorescence (Cy5) ensures the product formation. b) Time-dependent CLSM images depict the product diffusion from the PC-core to the surroundings, leading to the decrease of the magenta color at the core. c) The overall change in Cy5 fluorescence, measured at ROI-1, during the catalytic product formation and release. The green, and magenta channels correspond to $Atto_{488}$ and Cy5, respectively. Scale bar: 3 µm. Condition: [Dz⊂PCs] = 7 µM, [Subs-1] = 16.6 µM, TE buffer at pH 8, 50 mM $MgCl_2$ at 37 °C.

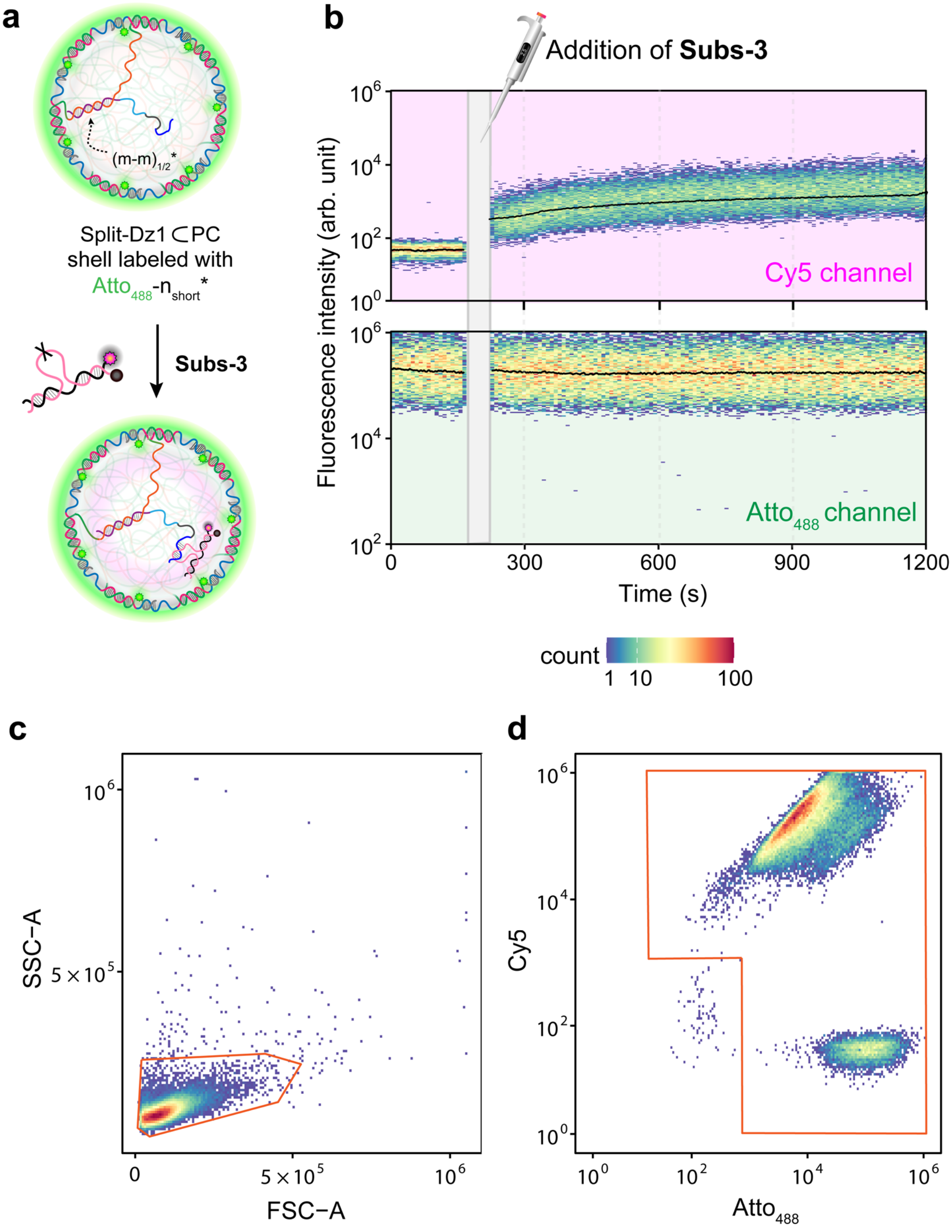

Supplementary Figure 8: protocellular (control experiment) $Atto_{488}$ and Cy5 fluorescence were monitored over time by flow cytometry. a) Schematic representation of dormant PC (shell labeled with $Atto_{488}$-n_{short}*) and Subs-3 loop binding. b) The time-dependent fluorescence intensity ($Atto_{488}$ and Cy5) of each PC is measured using flow cytometry. The slight increase at the Cy5 channel represents the residual fluorescence of the quenched Subs-3. The line shows the moving median. c) and d) the gating of the PCs in Subs-3 kinetics presented in Figure 4d, e. The green, and magenta channels correspond to $Atto_{488}$ and Cy5, respectively. Condition: [Split-Dz1⊂PC] = 0.5 µM and [Subs-3] = 1.5 µM in TE buffer at pH 8 at 25 °C.

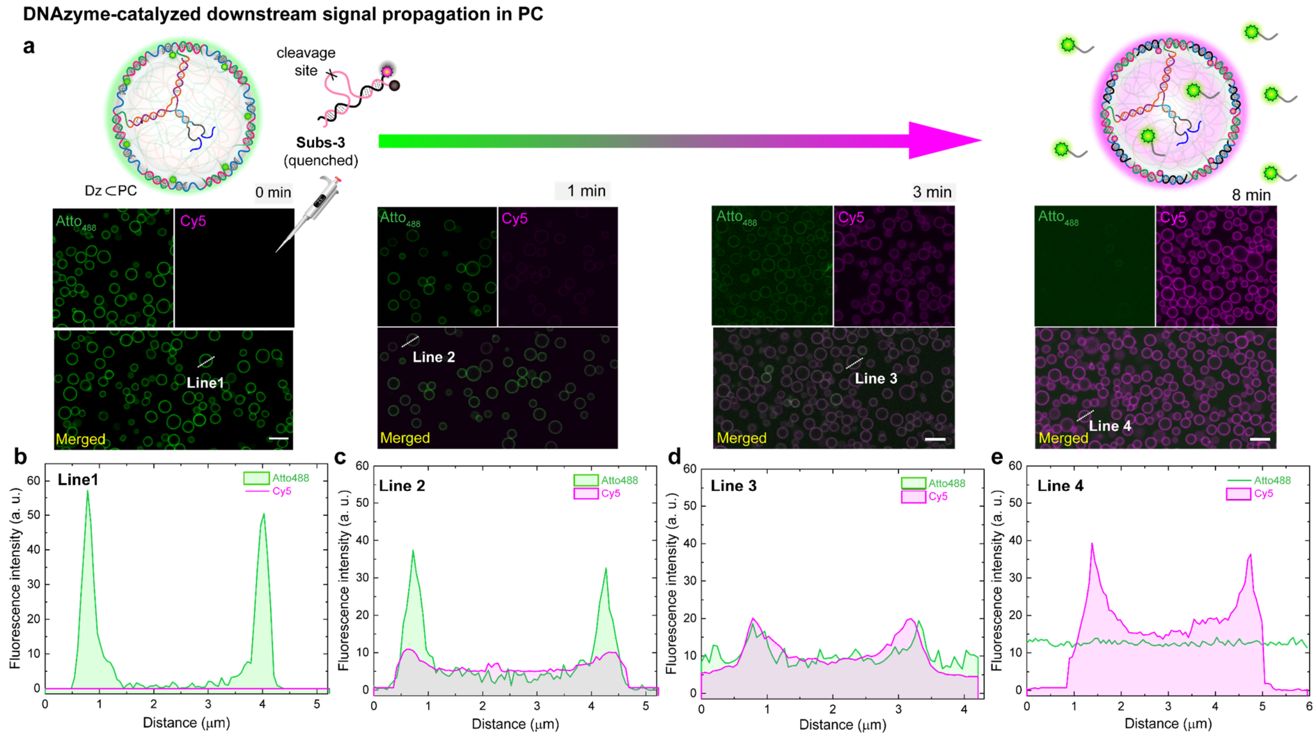

Supplementary Figure 9: CLSM instigation of DCBC, downstream BCDM, and DSD reaction in an active PC system. a) Time-dependent CLSM images of metabolic phenotype change in the Dz⊂Pcs. The shell of Dz⊂PCs are labeled with Atto$_{488}$-n$_{short}$* (green channel). At t=0 (before the addition of Subs-3), the PCs are visualized as empty green circles. Upon Subs-3 addition, a gradual increase of magenta fluorescence is observed at the shell of the PCs substituting the green fluorescence. b) to e) The line segment analysis on an active Dz⊂Pcs before (Line-1) and after (Line-2,3,4) the reaction and downstream transformation at different time points during the reaction. Scale bars: 5 µm. The concentration of Dz encapsulated is 7 µM and [Subs-3] = 15 µM at 37 °C. The green, and magenta channels correspond to Atto$_{488}$ and Cy5, respectively.

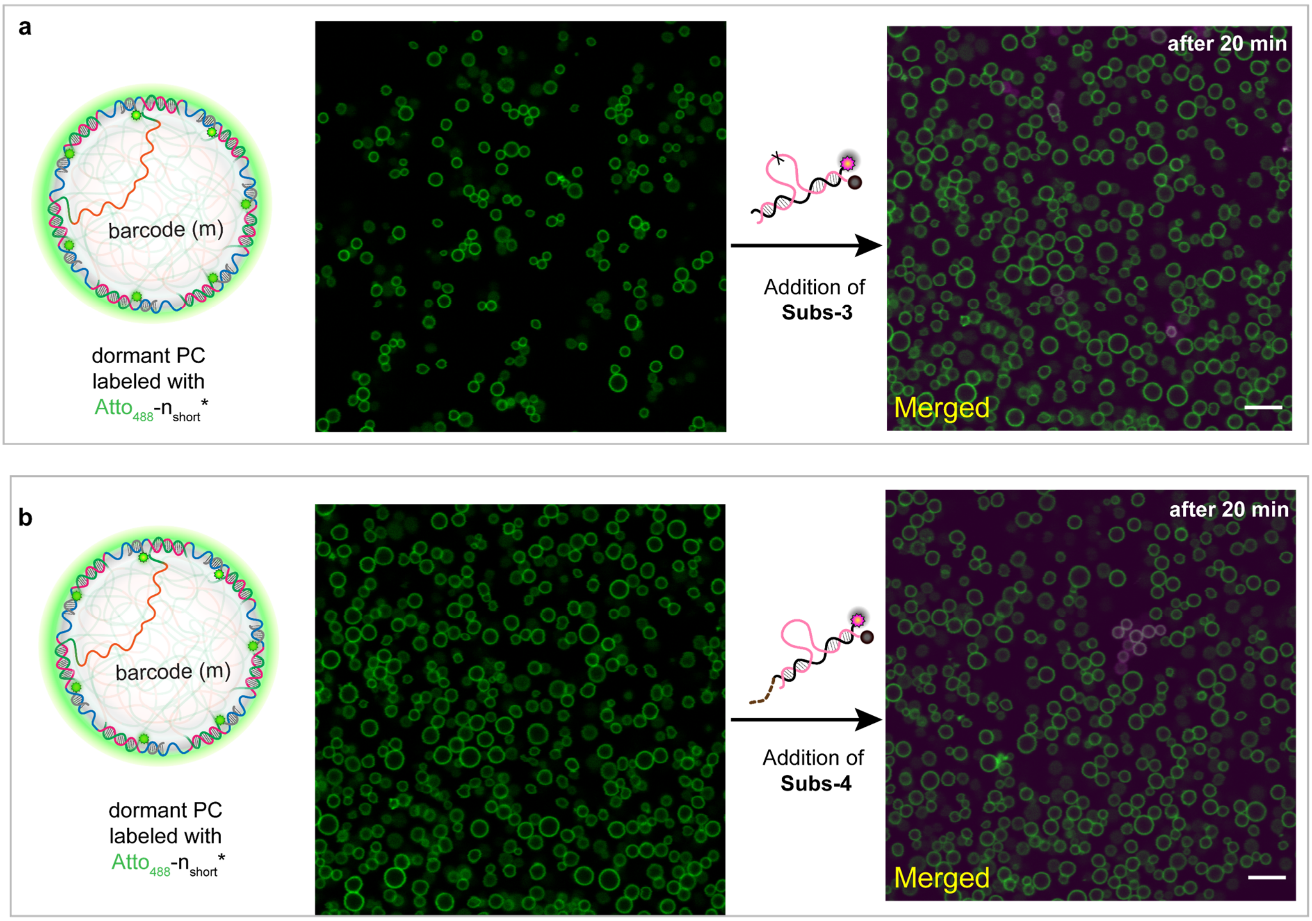

Supplementary Figure 10: CLSM investigation of dormant PCs in the presence of Subs-3 and Subs-4. a) CLSM images of before and after (20 min) the addition of Subs-3 (20 µM, 10 equivalent of shell barcode n) into a dispersion of dormant PCs, respectively. The PC shell is labeled with $Atto_{488}$-n_{short}* (green channel). b) CLSM images of before and after (20 min) the addition of Subs-4 (20 µM, 10 equivalent of shell barcode n) into a dispersion of dormant PCs, respectively. The PC shell is labeled with $Atto_{488}$-n_{short}* (green channel). In both cases, ~5 % leakage of the loop was observed. Scale bar = 5 µm. The CLSM images on the right represent green ($Atto_{488}$) and magenta (Cy5) merged channels.

The Prototissue size vs. Palindrome Density at the PC-Shell

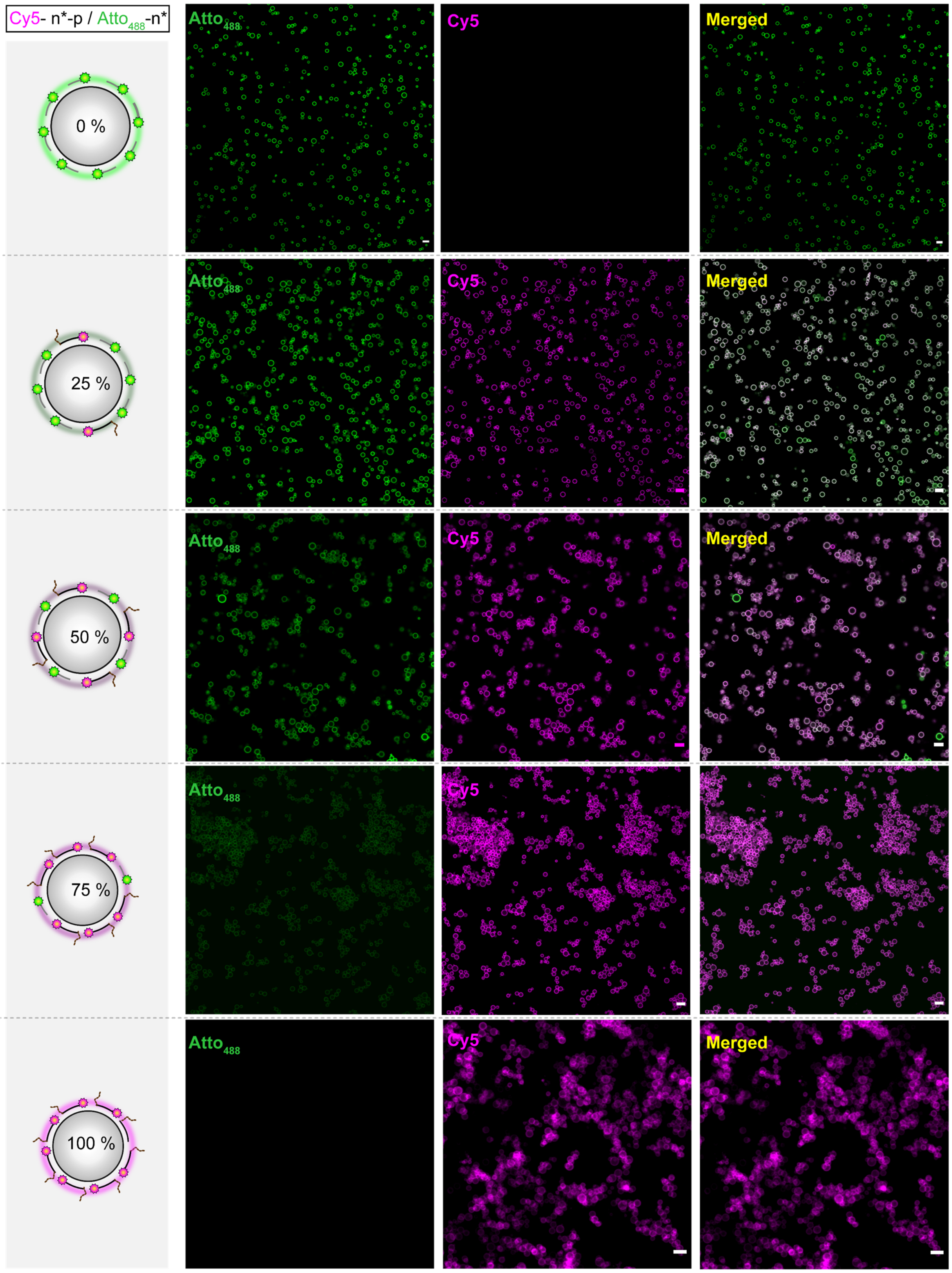

Supplementary Figure 11: The correlation between prototissue size and the surface density of the palindromic strand. The CLSM images of prototissue from five PC mixtures, in which the palindromic density at the PC-shell is varied from 0 to 100%. The ratio of the Cy5-n*-p (magenta) and Atto488-n* (green) is varied in 0, 25, 50, 75 and 100%. As Cy5-n*-p is the only one responsible for PC crosslinking, the prototissue size increases with the palindromic density at the PC-shell. The merged channel represents the fluorescence from both $Atto_{488}$ (green) and Cy5 (magenta). Scale bars: 5 µm.

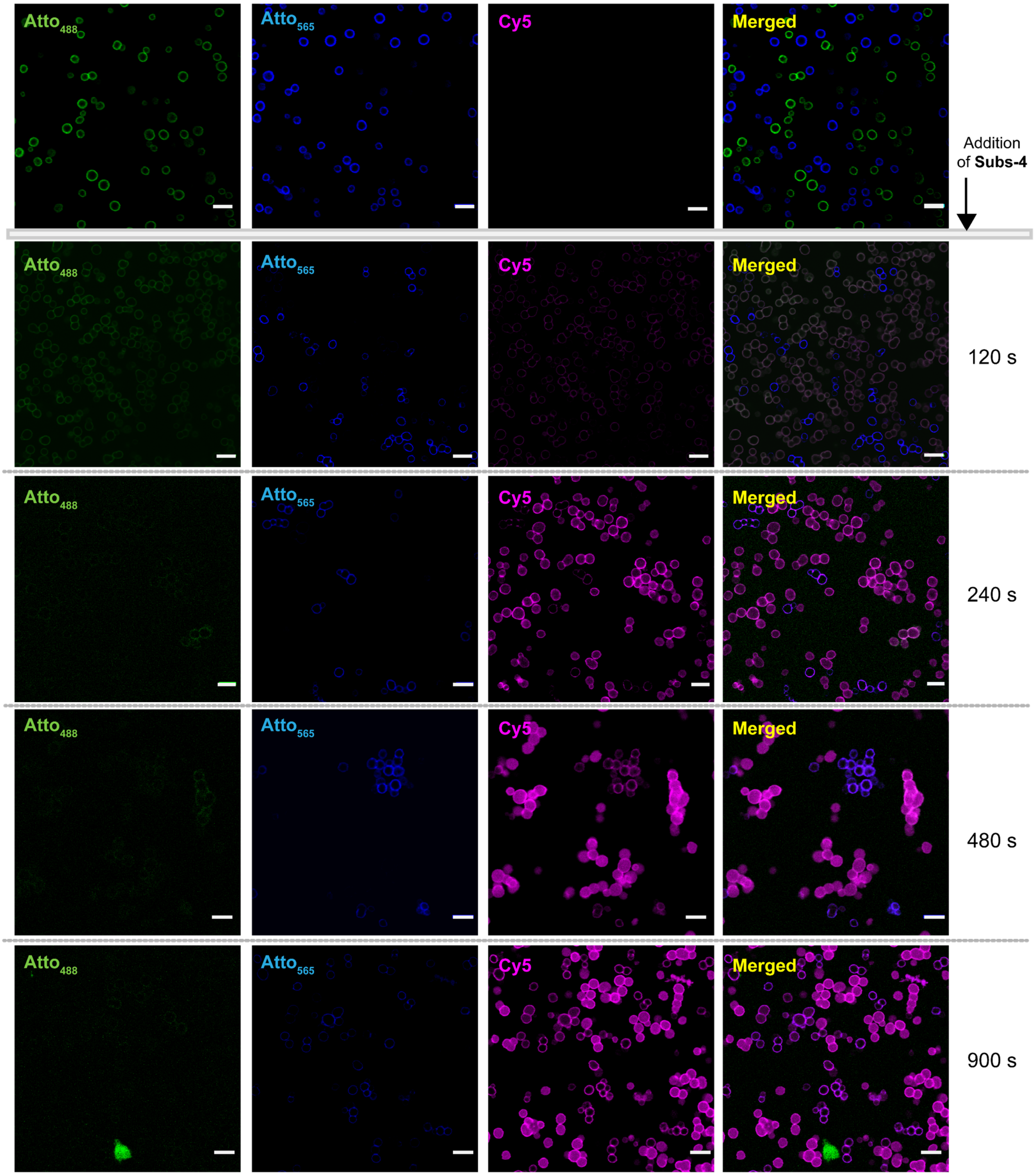

Supplementary Figure 12: Metabolic downstream interprotocellular communication and prototissue growth. The entire time-dependent CLSM image sets (with all the individual channels) of metabolic interprotocellular communication and the growth of prototissues in the mixed PC system. At *t*=0 (before the addition of Subs-4), the active and dormant PCs are visualized as empty green and blue spheres. Upon Subs-4 addition, a gradual increase of magenta fluorescence is observed at the shell of the active green PCs substituting the green fluorescence leading to the formation of sender prototissue. The dormant PCs' shell color (blue) starts decreasing while the magenta fluorescence increases, leading to the emergence of purple color within 4 min of the Subs-4 addition. The merged channel represents $Atto_{488}$ (green), $Atto_{565}$ (blue), and Cy5 (magenta) fluorescence. The scale bar = 5 µm. Condition: [Dz⊂PCs] = 5 µM, [Subs-4] = 35 µM in TE buffer at pH 8, 12.5 mM $MgCl_2$ at 35°C.

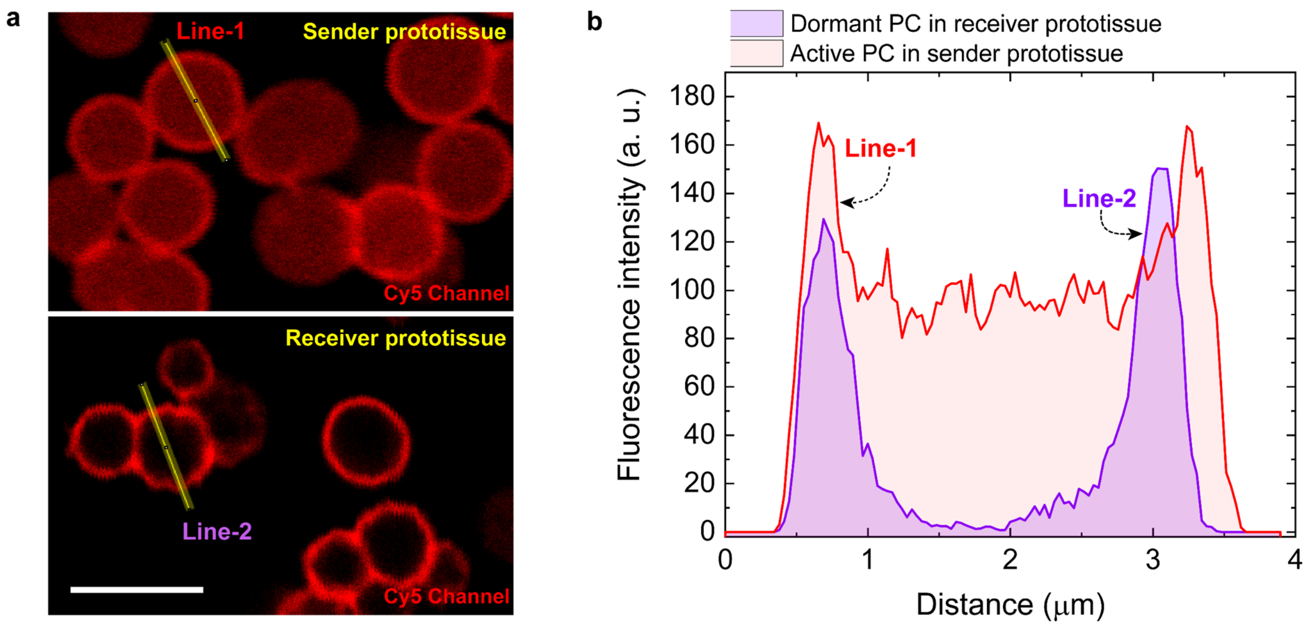

Supplementary Figure 13: The line-segment analysis on the Sender and receiver prototissue. a) The CLSM images of Sender (composed of active PC) and receiver (composed of dormant PCs) prototissue represented only the Cy5 channel (red). b) Line-segment analysis of Line-1 and Line-2 for Cy5 fluorescence intensity.

Supplementary References

[1] B. R. Wolfe, N. J. Porubsky, J. N. Zadeh, R. M. Dirks and N. A. Pierce, *J. Am. Chem. Soc.* **2017**, *139*, 3134-3144.
