# Supplementary material for: Signal-processing and adaptive prototissue formation in metabolic DNA protocells
Source: Nat Commun. 2022 Jul 8;13:3968. doi: 10.1038/s41467-022-31632-6 (PMC9270428; doi:10.1038/s41467-022-31632-6)
Supplement: Supplementary file 3 — Description of Additional Supplementary Files [file 41467_2022_31632_MOESM3_ESM.pdf]

Supplementary Video 1

Fluorescence recovery after successive photobleaching in the case of Subs-1 catalysis in DNAzyme-loaded protocells.
