# Supplementary material for: Signal-processing and adaptive prototissue formation in metabolic DNA protocells
Source: Nat Commun. 2022 Jul 8;13:3968. doi: 10.1038/s41467-022-31632-6 (PMC9270428; doi:10.1038/s41467-022-31632-6)
Supplement: Supplementary file 5 — Reporting Summary [file 41467_2022_31632_MOESM5_ESM.pdf]

nature portfolio

Corresponding author(s): Dr. Avik Samanta, Prof. Andreas Walther

Last updated by author(s): Jun 16, 2022

Reporting Summary

Nature Portfolio wishes to improve the reproducibility of the work that we publish. This form provides structure for consistency and transparency in reporting. For further information on Nature Portfolio policies, see our Editorial Policies and the Editorial Policy Checklist.

Statistics

For all statistical analyses, confirm that the following items are present in the figure legend, table legend, main text, or Methods section.

n/a	Confirmed	
☐	☒	The exact sample size (n) for each experimental group/condition, given as a discrete number and unit of measurement
☐	☒	A statement on whether measurements were taken from distinct samples or whether the same sample was measured repeatedly
☒	☐	The statistical test(s) used AND whether they are one- or two-sided<br>*Only common tests should be described solely by name; describe more complex techniques in the Methods section.*
☒	☐	A description of all covariates tested
☒	☐	A description of any assumptions or corrections, such as tests of normality and adjustment for multiple comparisons
☐	☒	A full description of the statistical parameters including central tendency (e.g. means) or other basic estimates (e.g. regression coefficient) AND variation (e.g. standard deviation) or associated estimates of uncertainty (e.g. confidence intervals)
☒	☐	For null hypothesis testing, the test statistic (e.g. F, t, r) with confidence intervals, effect sizes, degrees of freedom and P value noted<br>*Give P values as exact values whenever suitable.*
☒	☐	For Bayesian analysis, information on the choice of priors and Markov chain Monte Carlo settings
☒	☐	For hierarchical and complex designs, identification of the appropriate level for tests and full reporting of outcomes
☒	☐	Estimates of effect sizes (e.g. Cohen's d, Pearson's r), indicating how they were calculated

Our web collection on statistics for biologists contains articles on many of the points above.

Software and code

Policy information about availability of computer code

Data collection	Tecan Spark Platereader: Tecan SparkControl (SPARK control v3.1)<br>Leica Stellaris5 Microscope: (LAS X, v4.3.0.24308)<br>Gallios flow cytometer (v1.2, Beckman Coulter)<br>ScanDrop (Jena Analytic, FlashSoftPRO v1.2) spectrophotometer.<br>Intas Chemostar Touch 21.5 Gel Imager.<br>Thermocycler, Eppendorf: Mastercycler® nexus X2.
Data analysis	All numerical data was analyzed and visualized with Origin 2018, b9.5.0.193, Academic (OriginLab).<br>For all microscopy data, FIJI (ImageJ) version 1.52p was used. (https://imagej.net/Fiji).<br>In the cytometry, acquired data concatenated and gated using FlowJo (v10.6.1, Becton, Dickinson and Company, Franklin Lakes, NJ). Binned medians were calculated over 4 s of acquisition time, and data were plotted using ggCyto in R 4.1.1.

For manuscripts utilizing custom algorithms or software that are central to the research but not yet described in published literature, software must be made available to editors and reviewers. We strongly encourage code deposition in a community repository (e.g. GitHub). See the Nature Portfolio guidelines for submitting code & software for further information.

Data

Policy information about availability of data

All manuscripts must include a data availability statement. This statement should provide the following information, where applicable:

- Accession codes, unique identifiers, or web links for publicly available datasets
- A description of any restrictions on data availability
- For clinical datasets or third party data, please ensure that the statement adheres to our policy

A single excel file containing the source data of Figures 2c, 2i-k, 3b-c, 3g-h, 3j, 4c, 4i-l, and 5d-e is available with the paper. The source file also contains the numerical values of the number of protocells involved in flow cytometry kinetics, presented in Figures 2f-g and 4d-e. A data availability statement has been added to the manuscript. Additional supporting data are available from the corresponding author upon request.

Human research participants

Policy information about studies involving human research participants and Sex and Gender in Research.

Reporting on sex and gender	n/a
Population characteristics	n/a
Recruitment	n/a
Ethics oversight	n/a

Note that full information on the approval of the study protocol must also be provided in the manuscript.

Field-specific reporting

Please select the one below that is the best fit for your research. If you are not sure, read the appropriate sections before making your selection.

☒ Life sciences ☐ Behavioural & social sciences ☐ Ecological, evolutionary & environmental sciences

For a reference copy of the document with all sections, see nature.com/documents/nr-reporting-summary-flat.pdf

Life sciences study design

All studies must disclose on these points even when the disclosure is negative.

Sample size	For all fluorescence intensity measurements, two (or three) independent PC samples were measured in parallel. The error bars (Fig 2c,d and Fig. 4c) represent the standard deviation of the relative fluorescence. For the microscopic analysis, corresponding changes over a large area with ~40-50 PCs were presented. The reproducibility of these experiments was checked with two different PC batches and by imaging them using the same experimental parameters. Different areas were imaged in the kinetics to avoid the influence of photobleaching. For the statistical analyses of the prototissue formation, a box plot exhibiting a five-number summary of the data set was presented. The whiskers represent the standard deviation of at least 10 prototissue counts. For statistical analysis in Fig. 5d, e, average 400 PCs were counted for each palindromic density (details are in the source file).
Data exclusions	For all microscopy experiments, protocells that are not in the focal point were not included in the quantitative analyses. Protocells that drift out of the field of view of focal plane were excluded for the analysis. In the flow cytometry experiments, protocells without Atto488 and Cy5 fluorescence were excluded from the analysis (<3% of all protocells).
Replication	No technical replicates was reported in the paper. For the fluorescence intensity measurements, all the experiments were done in duplicates (in some cases in triplicates). For microscopic analysis, always multiple region of interests (ROIs) were imaged from a same sample. The reproducibility of the micrographs also checked using two different PC batches. We always presented more than 40 PCs per image at each time stamp. Cytometry kinetics were also checked in duplicates with two different PC batches. It is always recommended to use freshly prepared PC mixture for kinetic experiments to avoid aggregation.
Randomization	No randomization was used, since all the experiments were done independently with duplicates. Samples were not separated in groups.
Blinding	No randomization was used, since all the experiments were done independently and they were not separated in groups.

Reporting for specific materials, systems and methods

We require information from authors about some types of materials, experimental systems and methods used in many studies. Here, indicate whether each material, system or method listed is relevant to your study. If you are not sure if a list item applies to your research, read the appropriate section before selecting a response.

Materials & experimental systems

n/a	Involved in the study
☒	☐ Antibodies
☒	☐ Eukaryotic cell lines
☒	☐ Palaeontology and archaeology
☒	☐ Animals and other organisms
☒	☐ Clinical data
☒	☐ Dual use research of concern

Methods

n/a	Involved in the study
☒	☐ ChIP-seq
☐	☒ Flow cytometry
☒	☐ MRI-based neuroimaging

Flow Cytometry

Plots

Confirm that:

☒ The axis labels state the marker and fluorochrome used (e.g. CD4-FITC).

☒ The axis scales are clearly visible. Include numbers along axes only for bottom left plot of group (a 'group' is an analysis of identical markers).

☒ All plots are contour plots with outliers or pseudocolor plots.

☒ A numerical value for number of cells or percentage (with statistics) is provided.

Methodology

Sample preparation	PC sample preparation is described in the Methods section.
Instrument	Gallios flow cytometer (Beckman Coulter). Atto488 and Cy5 were excited with a 488-nm or 638-nm laser and detected using a 525/40-nm or 660/20-nm bandpass filter, respectively.
Software	Gallios Cytometer (v1.2, Beckman Coulter) software was used for data aquisition. Acquired data before and after the addition of the substrate was concatenated and gated using FlowJo (v10.6.1, Becton, Dickinson and Company). Binned medians were calculated over 4 s of acquisition time, and data were plotted using ggCyto (https://doi.org/10.1093/bioinformatics/bty441) in R 4.1.1 (https://www.r-project.org).
Cell population abundance	In every cytometry kinteics presented in the manuscript, approx. 200 protocells were measured per second.
Gating strategy	PCs were identified in the FSC / SSC scatter plot. PCs without Atto488 and Cy5 fluorescence were excluded from the analysis (<3% of all PCs). The gating strategy is depicted in Figure 2e.

☒ Tick this box to confirm that a figure exemplifying the gating strategy is provided in the Supplementary Information.
